# Hydration Characteristics and Early Strength Evolution of Classified Fine Tailings Cemented Backfill

**DOI:** 10.3390/ma16030963

**Published:** 2023-01-20

**Authors:** Yunpeng Kou, Yuchao Deng, Yuye Tan, Chongchong Han, Weidong Song

**Affiliations:** 1 Key Laboratory of the Ministry of Education for High Efficient Mining and Safety in Mental Mines, Beijing 100083, China; 2 School of Civil and Resource Engineering, University of Science and Technology Beijing, Beijing 100083, China; 3 Backfill Engineering Laboratory Branch of Shandong Gold Mining Science and Technology Co., Ltd., Yantai 261400, China

**Keywords:** classified fine tailings, porosity, hydration reaction, volume resistivity, early strength

## Abstract

To explore the hydration characteristics and early strength evolution of classified fine tailings cemented backfill (CFTCB), a nuclear magnetic resonance (NMR) analysis and a volume resistivity test were performed on classified fine tailings filling slurry (CFTFS). The early hydration products of CFTCB were studied by scanning electron microscopy (SEM) and X-ray diffraction (XRD) phase analysis. Uniaxial compressive strength (UCS) test was carried out, and the microscopic characteristics and strength rules of the hydration reaction of CFTCB were analyzed. Based on the experiment, we found the law of water content change and porosity evolution. The early hydration reaction can be divided into the dissolution, setting, and hardening stages. The volume resistivity test results show that the volume resistance of filling slurry increases slowly at first then decreases, and finally increases rapidly. The variation trend of volume resistivity is related to the degree of hydration reaction. When combined with the hydration characteristics of backfill materials, the hydration reaction rate determines the growth rate of early strength of backfill, and the formation of hydration products is the reason for the early strength increase in backfill. The research conclusion has an important theoretical guiding value and engineering significance in mine filling production organization and filling ratio parameter optimization.

## 1. Introduction

At present, underground gold mines usually adopt the classified coarse tailing cemented filling approach in goaf treatment [1]. Classified fine tailings are discharged into the tailings pond for storage; thus, the tailings dam faces collapse, landslide, dam breakage, and other safety hazards. Mine tailings discharge has become a “choke” problem restricting mine green development due to the high mineral rate and the tight safety and environmental protection situation. With the rapid development of high-concentration filling technology, a new cementitious material suitable for fine tailings filling has been successfully developed. Moreover, the demand for sand in building materials and other industries is high, and coarse tailings have a good market demand after dehydration. Under the support of a series of policies related to the construction of tailing-free mines in China, constructing green tailing-free mines has become a new idea and approach to fill underground goafs with classified fine tailings and use classified coarse tailings in building materials. The filling application of fine tailings is still limited at home and abroad. When classified fine tailings are used as fillers, the requirement for the early strength of the backfill is high. The backfilling laboratory of the Shandong Gold Group has conducted numerous beneficial research on fine tailings and developed a binder suitable for fine tailings [2]. However, the early strength evolution characteristics of fine cemented tailing backfill require further study.

The use of classified fine tailings as a filling aggregate is a relatively advanced attempt and practice at home and abroad, and classified fine tailings’ full hydration reaction with cementing materials is a crucial link in strength formation. A previous study [3] shows that the essence of the hardening diagenesis of filling slurry is the continuous hydration of the cementitious materials to produce hydration products. When cement is used as a cementing material, the reaction with fine tailings cannot play the necessary curing effect [4]. Xu Wenbin [5] studied the influence of the type and shape of hydration products on the strength development of backfill. Guan Shiliang [6], FALL [7], and Qiu Jingping [8] found that the external factors affecting the strength of cemented backfill include the curing conditions and internal factors such as the cement–tailings ratio (CTR) and the slurry concentration. FALL [9] conducted mechanical properties and microstructure tests on backfill samples at different curing times and temperatures and found that the degree of cement hydration reaction decreases in low-temperature environments. Deng Xuejie [10] found that the larger the particle size is, the higher the number of hydration products will be. Moreover, some studies have pointed out that the particle size of tailings has an important influence on the porosity and internal pore size distribution of backfills, thus affecting the backfill strength [11]. In addition, to study the formation mechanism of backfill strength, domestic and foreign scholars have performed many experiments. Gan Deqing [12] found that the UCS of super-fine tailings backfill is higher than that of classified fine tailings backfill because Ca(OH)_2_ is rarely generated in the hydration process of ultra-fine tailings, the calcium silicate hydrate (C-S-H) gel content is high, and the hydration products and morphology of the two are different. The results of previous research [13] show that the CTR has a significant effect on the resistivity and heat of hydration. The tailings content determines the overall resistivity value, and the heat of hydration is positively correlated with the cement content. The resistivity and heat of hydration can more fully reveal the hydration reaction and microstructure evolution. Wei Dingyi [14] found that the influencing factors of the hydration heat of tailings cemented backfill are the slurry concentration, the CTR, and the binder dosage. Liu Shulong [15] used blast furnace slag instead of part of cement as cementing material to study the early strength characteristics of full tailings backfill, and found that compared with cement as cementing material, the backfill had higher early strength, but did not study the mechanism of slag in hydration reaction.Peng Xiaopeng [16] found that the pore water content and characteristics in the cemented tailings backfill material affect the migration of the hydration and external sulfur ions of the cementitious material, thus affecting the strength of the backfill. Jia Shijie [17] and S.K. Behera [18] found that the fly ash content can reduce the early strength of cemented backfill and explained the reason from the physicochemical perspective. Tao Boshi [19] found that the main hydration products of fiber-cement-based cemented backfill are ettringite (Aft), C-S-H gel, and Ca(OH)_2_, and the addition of fiber enhances the ability of the hydration products to resist external deformation, enabling the filling body to maintain good mechanical properties. Li Wenchen [20] and Benzaazoua M. [21] found that sulfate ions have an inhibitory effect on cement hydration, the C-S-H adsorption of sulfate decreases the early backfill strength, and the higher the sulfate concentration is, the lower the early backfill strength is. The results of related research [22] show that the binder content has a profound effect on the short-term strength of the backfill. Hou Chen [23] found that the higher the binder content is, the stronger the hydration reaction of the tailings backfill is, and continuous hydration leads to the continuous development of strength.

However, the research on the law of water transformation and evolution in the hydration and hardening process of filling slurry and its internal influence on the early strength formation of backfills is limited. Through NMR, UCS, and SEM experiments, this paper studies the early-stage strength evolution law and stage characteristics of classified fine tailings backfill from the perspective of the hydration process and products of filling slurry, which has an important theoretical guiding value and an engineering significance in mine filling production organization and the optimization of the filling ratio parameter.

## 2. Experimental Materials and Scheme

### 2.1. Material of Experiment

#### 2.1.1. Classified Fine Tailings

**Figure 1:**
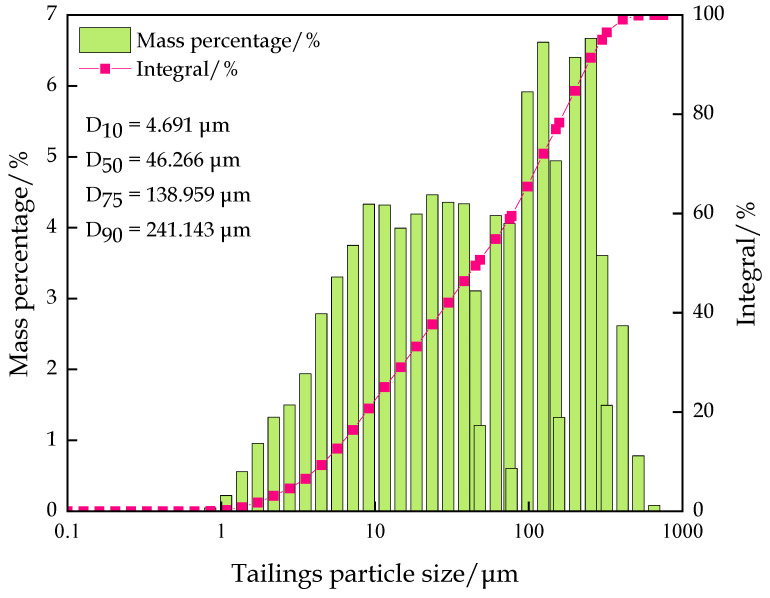
Particle size distribution diagram of classified fine tailings.

**Figure 2:**
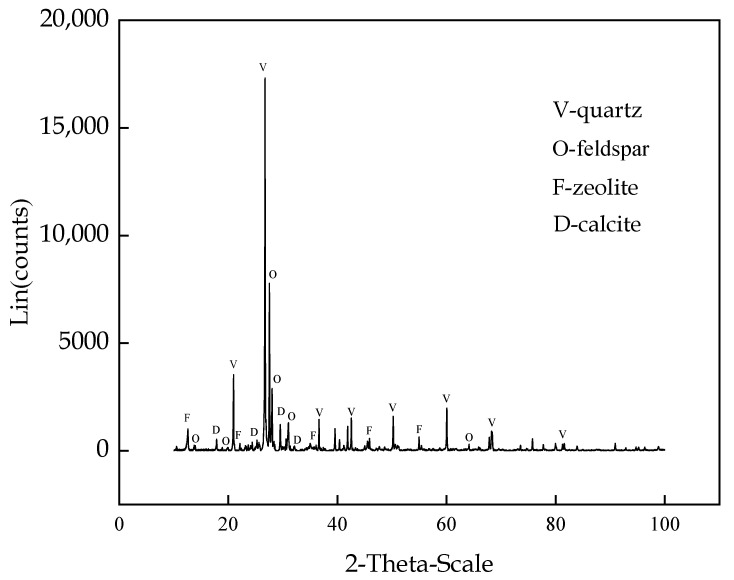
XRD phase analysis of classified fine tailings.

Classified fine tailings were selected for the experiment, and the experimental material was derived from the ore tailing of a gold mine in Shandong Province, China. The classified fine tailings are dried before configuring the filling slurry. The physical parameters are presented in Table 1, and the main chemical components are listed in Table 2. The grain size is shown in Figure 1. The content of 0–45 µm tailings is approximately 50%, and the content of tailings with sizes less than 300 µm, which are considered fine-grained tailings, is approximately 95%. As shown in Figure 2, the composition of tailings is relatively simple, mainly composed of quartz, feldspar, zeolite, and calcite. The classified fine tailings, which are mainly composed of SiO_2_ and Al_2_O_3_, are a good inert filling material suitable for downhole filling raw materials.

#### 2.1.2. Cementitious Material

**Figure 3:**
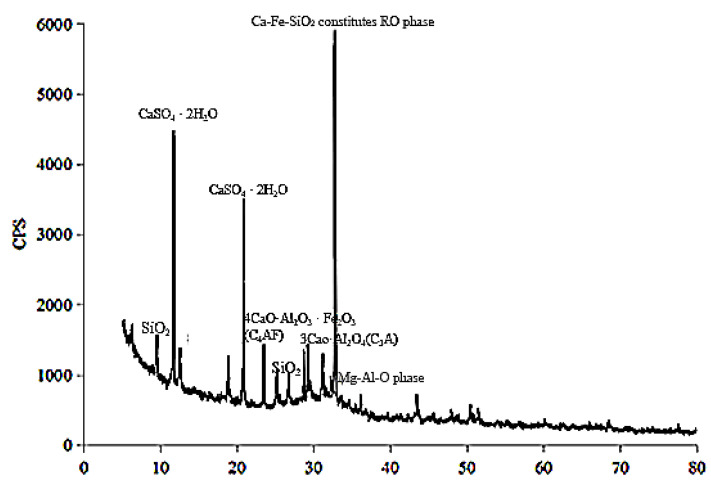
XRD phase analysis of cementitious material.

Cementitious material, “filling C material”, which was independently developed by a laboratory, was used in this experiment. The mineral composition of the material is mainly slag and gypsum. The chemical composition of the filling C material is shown in Table 3, and its chemical composition is mainly CaO, SiO_2_, and Al_2_O_3_. As shown in Figure 3, these substances formed tricalcium silicate 3CaO-SiO_2_(C_3_S), tricalcium aluminate 3CaO-Al_2_O_3_(C_3_A), and gypsum CaSO_4_·2H_2_O, etc. This material, which contains small amounts of MgO, Fe_2_O_3_, and TiO_2_, is a kind of active cementitious material suitable for underground filling raw materials. In addition, the existence of slag in “filling C material” reduces the preparation cost.

### 2.2. Experimental Scheme

#### 2.2.1. NMR Analysis of Hydration Reaction

The porosity of the sample and the content and type of water in the sample was determined by an NMR analytical system (MesoMR23-060H-I, Suzhou, China). A slurry with a slurry concentration of 64% (The sum of the mass of tailings and filling C material divided by the total mass of slurry is the slurry concentration, and the water/solid ratio is 36:64) was prepared at CTRs of 1:4, 1:6, and 1:8. Three samples were prepared for each CTR, and the CFTFS was evenly stirred into a 30 mL lidded cylinder glass bottle. After curing for a certain number of hours (0.5, 1, 1.5, 2, 4, 6, 10, 18, 24, 30, 34, 42, 48, and 72 h) at a constant temperature in a humidity-curing box, NMR analysis was performed.

#### 2.2.2. Resistivity Experiment of CFTFS during Hydration Reaction

A resistance tester (ATSIO) was used to measure the resistivity of the CFTFS. The slurry concentration was 64%, and the CTR was 1:6. Three groups of samples were configured to test the volume resistivity of the samples according to the AC impedance method.

#### 2.2.3. Micro Analysis Experiment of Hydration Products

A Rigaku D/Max2500 X-ray diffractometer was used to determine the mineral composition of the cemented backfill samples. A sample with a concentration of 64% and a CTR of 1:6 was prepared, and the sample was measured at 6, 12, 24, and 48 h after preparation.

A JSM-6700F cold field emission scanning electron microscope was used to determine the microstructure of the cemented backfill samples. For backfill samples with a concentration of 64% and a CTR of 1:6, the phase characteristics of the hydration products at different early ages (3 and 7 days) were tested.

#### 2.2.4. UCS Test of CFTCB

**Figure 4:**
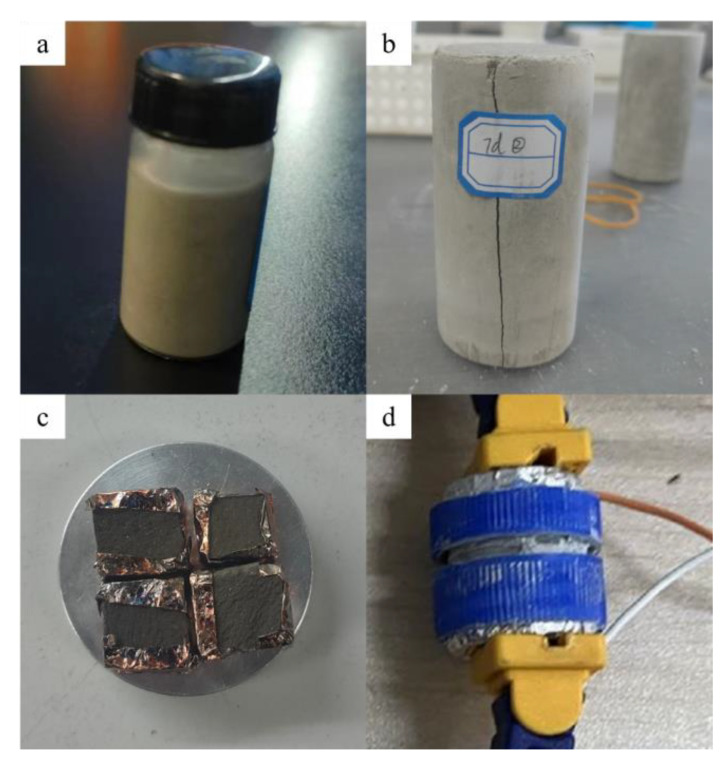
Schematic diagram of experimental materials: (**a**) CFTFS by NMR analysis; (**b**) CFTCB by UCS; (**c**) CFTCB by SEM; (**d**) CFTFS by volume resistivity experiment.

UCS test was performed on the CFTCB with a slurry concentration of 64% and a CTR of 1:6. Three groups of samples were prepared, and each group was prepared in three 50 mm × 50 mm × 100 mm cylinders. After curing for 2, 3, and 7 days at a constant temperature in a humidity curing box, the strength of the CFTCB was measured after curing the test block. Figure 4 shows the diagram of experimental materials for each experiment.

## 3. Experimental Results and Analysis

### 3.1. Characteristics of Hydration Process of CFTFS

#### 3.1.1. Variation Law of Porosity

**Figure 5:**
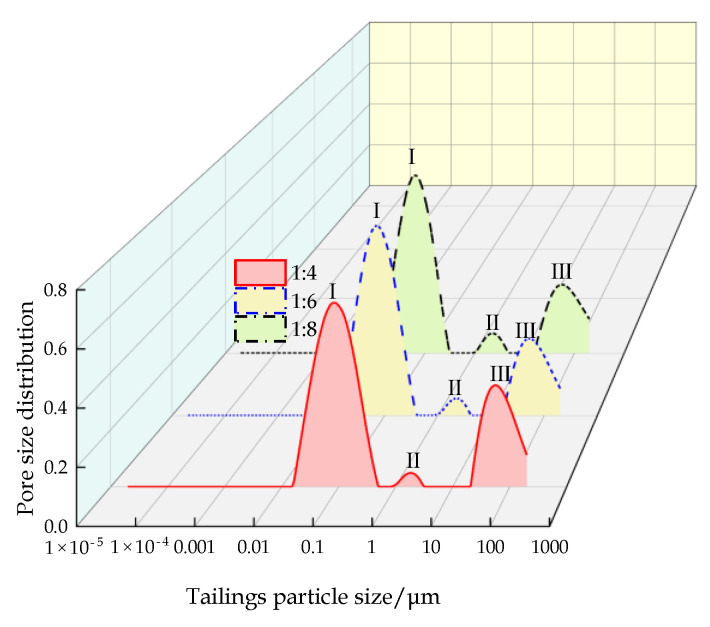
Pore size distribution of CFTFS.

The pore size distribution of the CFTFS formed after 72 h of hydration reaction at different CTRs is shown in Figure 5. The figure shows that the pore size distribution of the CFTFS contains one main peak and two secondary peaks, which are defined as peaks I, II, and III from left to right. The proportion of peak area represents the proportion of pores, and the proportion of the peak area of peak I is approximately 70%, indicating that the pore distribution of the CFTFS is mainly concentrated in the 0.01–1 µm range. As shown in the figure, the peak value of peaks I and II and the area proportion decrease with the increase in the CTR; the peak value of peak III and the area proportion increase, indicating that the pore size of the CFTFS and the number of large pores increase with the CTR.

**Figure 6:**
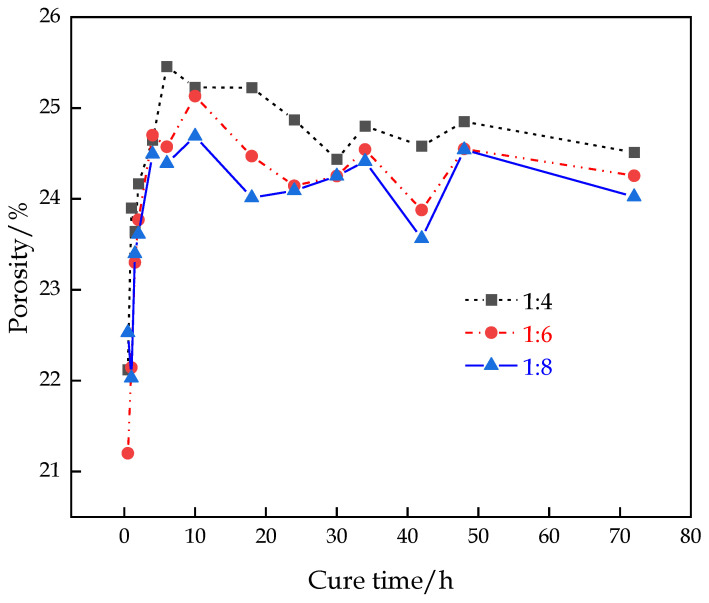
Variation curve of porosity of CFTFS over time.

The variation in porosity in the hydration process of the CFTFS with different CTRs is shown in Figure 6. With the increase in the curing time, the porosity of the CFTFS shows a rapidly increasing trend at first, followed by a slight decrease, and finally stabilizes at approximately 24%. The decrease in the porosity directly shows the process of backfilling the sample from loosely to densely and reflects the generation of hydration products and the nucleation crystallization mode. The porosity of the CFTFS increases with the CTR.

#### 3.1.2. Variation Law of Water Type and Content

**Figure 7:**
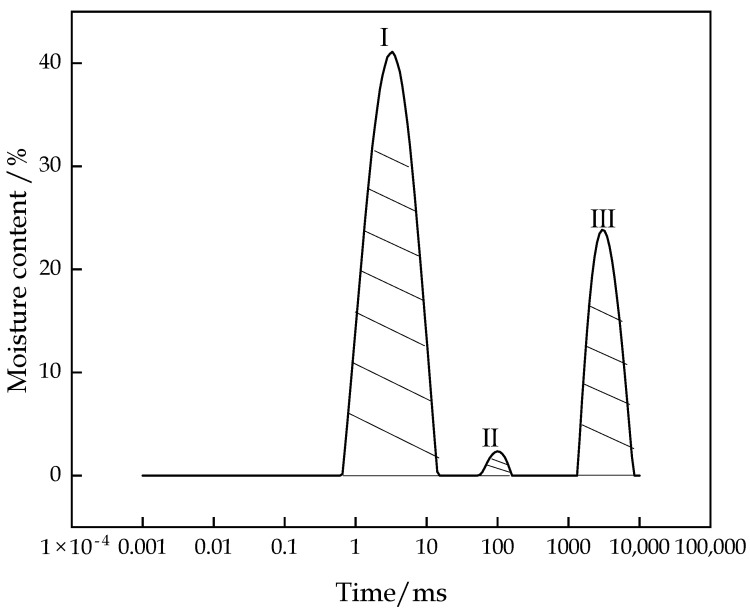
T2 spectral inversion curve.

The variation trend of the T2 spectrum distribution of the CFTFS with a CTR of 1:6 within 0–10,000 ms is shown in Figure 7. The figure shows that the T2 spectrum includes three relaxation peaks, which are denoted as peaks I, II, and III from left to right. The distribution of each peak is as follows: Peak I ranges from 0.65 ms to 14.15 ms, Peak II ranges from 51.71 ms to 148.20 ms, and Peak III ranges from 1431.46 ms to 7842.82 ms. Each relaxation signal corresponds to a kind of water, so this experiment detected the existence of three different bound states of water in the CFTFS. The CFTFS has adsorbed water, chemically bound water, free water, and pore water. The results of previous research [24] show that the T2 value of chemically bound water is very transient, so the relaxation signal of the chemically bound water cannot be obtained by the NMR experiment. Therefore, the three relaxation peaks of the T2 spectral inversion curve correspond to the NMR signals of the hydrogen nuclei in the adsorbed water, the free water, and the pore water. Studies have shown that [25] the fluidity of water is positively correlated with the T2 value. Accordingly, peak I corresponds to the relaxation signal represented by the adsorbed water. Peak II corresponds to the relaxation signal represented by the pore water, and peak III corresponds to the relaxation signal represented by the free water. Figure 7 shows that peak–peak area I occupies the largest proportion. Adsorbed water is the main form of water in the CFTFS, with the adsorbed water content accounting for approximately 40%, followed by the free water and pore water contents.

**Figure 8:**
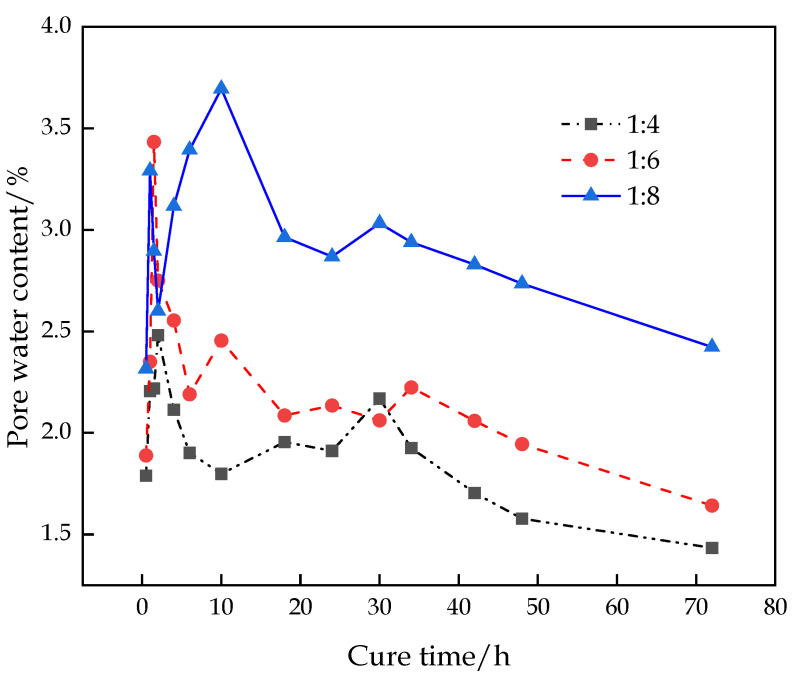
Characteristic curve of pore water moisture variation.

Figure 8 shows that the change in the pore water content of the CFTFS presents a trend of sharp fluctuation–rapid increase–slow decrease due to the settlement process of the CFTFS particles in the early stage, resulting in constant changes in particles and pores. The instrument test is only an instantaneous value, so it presents dramatic fluctuations in the early stage. Then, the intermolecular movement accelerates, the pore size enlarges, and the pore water content increases with the progress of the hydration reaction. When the hydration reaction reaches a certain degree, the hydration reaction rate and pore water content decrease.

#### 3.1.3. Stage Characteristics of Hydration Reaction

**Figure 9:**
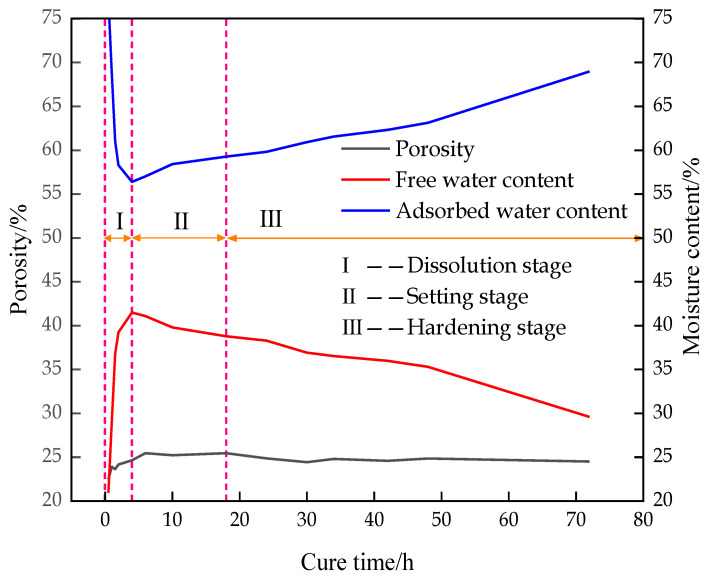
Characteristic curve of porosity and moisture content in different stages.

**Figure 10:**
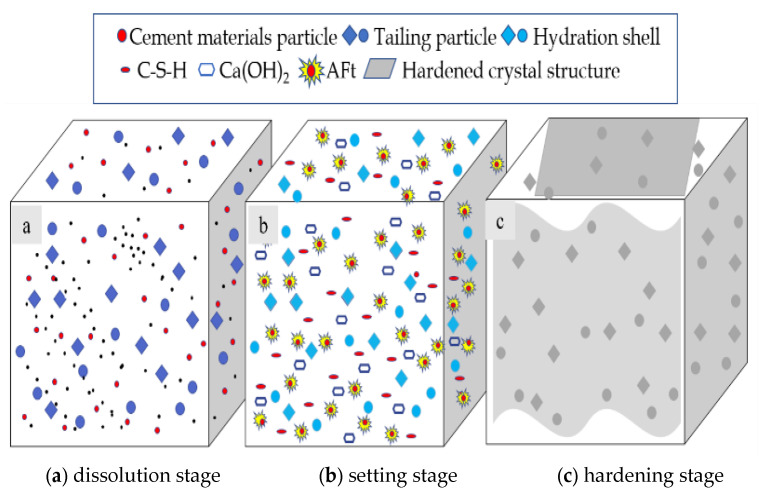
Schematic diagram of hydration feature description model.

To obtain the hydration characteristics of the CFTFS, the porosity of the CFTFS (CTR of 1:6) was combined with the variation curves of free water and adsorbed water, as shown in Figure 9. The figure shows a good correlation in different periods, which reflects the hydration characteristics. The hydration process can be divided into dissolution stage I, setting stage II, and hardening stage III. In the dissolution stage of the hydration process, the intermolecular movement is accelerated, the pores are enlarged, and the pore water content is increased with the drastic changes in the hydration reaction. Moreover, a part of the adsorbed water is separated from the tailings particles and gathered in the upper part of the sample bottle as free water due to the settlement of tailings particles, rapidly decreasing the adsorbed water content and rapidly increasing the free water content. During the setting and hardening stages of the hydration reaction, the free water separated from the upper part of the sample bottle reacts with the filling material again, resulting in a slow increase in adsorbed water and a decrease in free water. The specific analysis is as follows:

As shown in Figure 10, the hydration reaction of the CFTFS is divided into three stages: dissolution stage I, setting stage II, and hardening stage III.

(1) Dissolution stage I

After mixing the filling material with water and stirring evenly, the main components of the cementing material dissolve quickly. The surface of the classified fine tailings and other particles also show Al_2_O_3_, CaO, Fe_2_O_3_, and other dissolved substances. Then, many positive and negative charged ions are released into the water. Slag is composed of the active vitreous body of slag and a protective layer of silicon–oxygen network structure on its surface. In the alkaline environment, the OH^−^ ions in the solution react with the protective layer on the surface of the slag glass. After the protective layer is destroyed, OH^−^ ions enter the slag glass body and react with active CaO to form Ca(OH)_2_. Given the high activity of tricalcium aluminate (C_3_A) in the filling C material, C_3_A first reacts with gypsum under alkaline conditions. In the dissolution stage, the molar ratio of gypsum to C_3_A is generally greater than 3, and needle-like ettringite (Aft) is mainly formed after the reaction. 

The generation of Aft at this stage produces a wrapping film on the surface of C_3_A, thus decelerating the hydrolysis rate of C_3_A. At the late dissolution stage, the content of ions in the solution stabilizes temporarily, and the hydration reaction then proceeds to the setting stage.

(2) Setting stage II

With the continuous dissolution of each component in the filling C material and the active component in the classified fine tailings, at this time, the slag glass body disintegrates, and Ca(OH)_2_ reacts with Al_2_O_3_ and SiO_2_ to form C-S-H gel. This stage is mainly a hydration reaction process of the filling C material. The effect of the hydration of the filling C material on the classified fine tailings is generally regarded as the passing of hydration product C-H through the phase interface to form a hydration film on the surface of the tailing particles, as shown in Figure 10b.

(3) Hardening stage III

At the end of the setting period of the CFTFS, the hydration reaction rate decelerates until it stops, and the hydration products produced (flaky-like C-H, layered C-S-H and acicular Aft) increase the hydration compounds on the surface and surrounding of the classified fine tailings particles, and the particle spacing continuously narrows, thereby overcoming the repulsion between the suspended solids in the slurry. Under the action of the Van der Waals and chemical bonding forces, particles of different particle sizes polymerize with each other, or the crystal nuclei of particles connect with each other, thus forming a hardened crystalline structure and gradually condensing the backfill into a whole.

### 3.2. Variation in Resistivity of CFTFS

**Figure 11:**
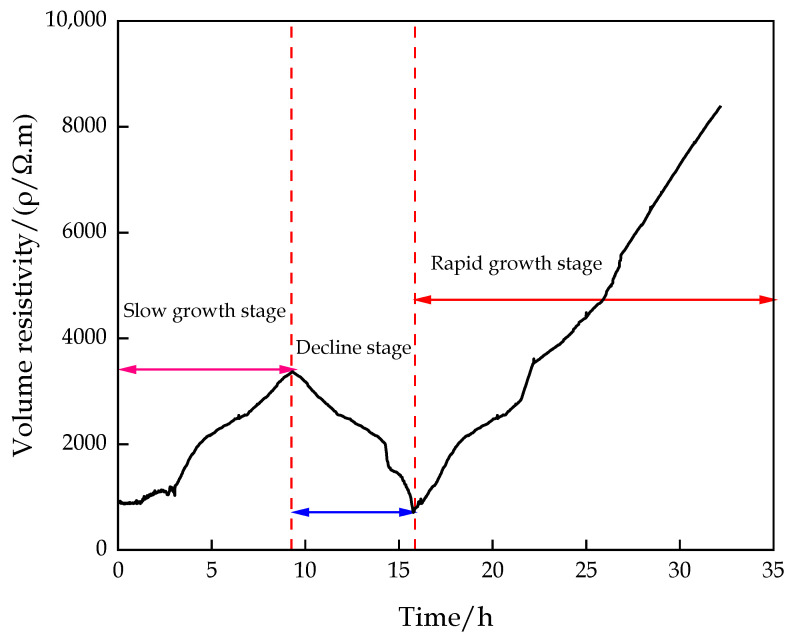
Phase variation characteristic curve of volume resistivity.

The change characteristics of the volume resistivity of the CFTFS with a CTR of 1:6 were divided into three stages, namely, slow growth stage I, decline stage II, and rapid growth stage III, as shown in Figure 11. The volume resistivity of the sample increases slowly first, decreases, and then increases rapidly over time. This trend can be explained by the increase in the CFTFS’s internal temperature after stirring the filling c material with water. When the temperature of the semiconductor material increases, such as the filling c material, the internal electron movement intensifies, and the resistance of the carrying current decreases, thus reducing the resistance of the material. In addition, after adding water to the filling c material, the soluble components in the filling c material dissolve, and mineral hydrolysis occurs, resulting in the increase in the ion concentration in the filling slurry and the enhancement of the electrical conductivity. In the first stage, the cementation process of the hydration reaction of the sample is dominant, and the generated hydration products cause the volume resistivity of the sample to increase slowly. In the second stage, the resistance and the volume resistivity decrease due to the increase in the ion concentration. In the third stage, the sample slurry gradually condenses with the progress of the hydration reaction, leading to the gradual increase in the internal resistance of the material, indicating that the resistivity increases with time. The hydration reaction stage is in the dissolution stage within 0–4 h, the setting stage within 4–18 h, and the hardening stage after 18 h. The volume resistivity increases slowly during the dissolution stage of the hydration reaction. During the hardening period, the volume resistivity increases rapidly. In particular, the volume resistivity in the setting phase of the hydration reaction shows three trends with the increase in time, depending on the degree of hydration reaction.

### 3.3. Hydration Products and Microstructure Characteristics

#### 3.3.1. Analysis of Hydration Products

**Figure 12:**
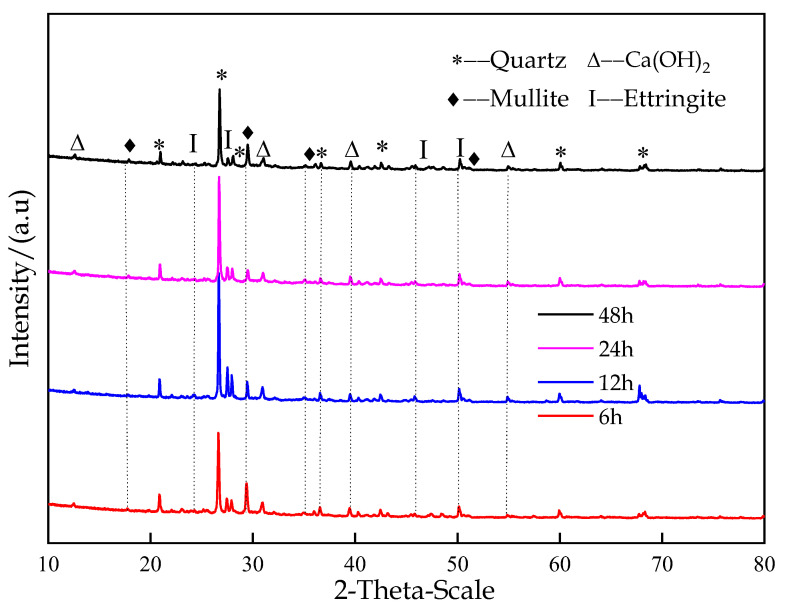
Formation of hydration products.

Figure 12 shows the XRD patterns of the classified fine tailings filling materials with a CTR of 1:6 at different curing times. According to the phase retrieval results, hydrated products, such as Ca(OH)_2_ and Aft, formed after the condensation of the classified fine tailings filling material. After curing for 6 h, Aft and Ca(OH)_2_ formed in small amounts. After 12 h, the relative contents of Aft and Ca(OH)_2_ increased, indicating that more hydration products were generated with the progress of the reaction. Ca(OH)_2_, which belongs to the tripartite crystal system, is the product of the hydration reaction, and the crystals mostly exhibit a flake-like structure. Aft is the product formed by the reaction of C_3_A in the silicate filling c material and the gypsum. It is worth noting that after the hydration reaction, mullite was detected by XRD. Mullite may come from tailings or filling C material, and it is not a hydration product. As the C-S-H gel is an amorphous phase, it cannot be detected by XRD, so SEM can be used for observation.

When combined with the division of the hydration reaction stages, the hydration reaction went through the dissolution and setting stages within 12 h, and numerous hydration products were generated. After 18 h, the hydration reaction entered the hardening stage and then stopped. The XRD results show that nearly no new hydration products were generated, and the backfill structure gradually stabilized.

#### 3.3.2. Microscopic Morphology Analysis

**Figure 13:**
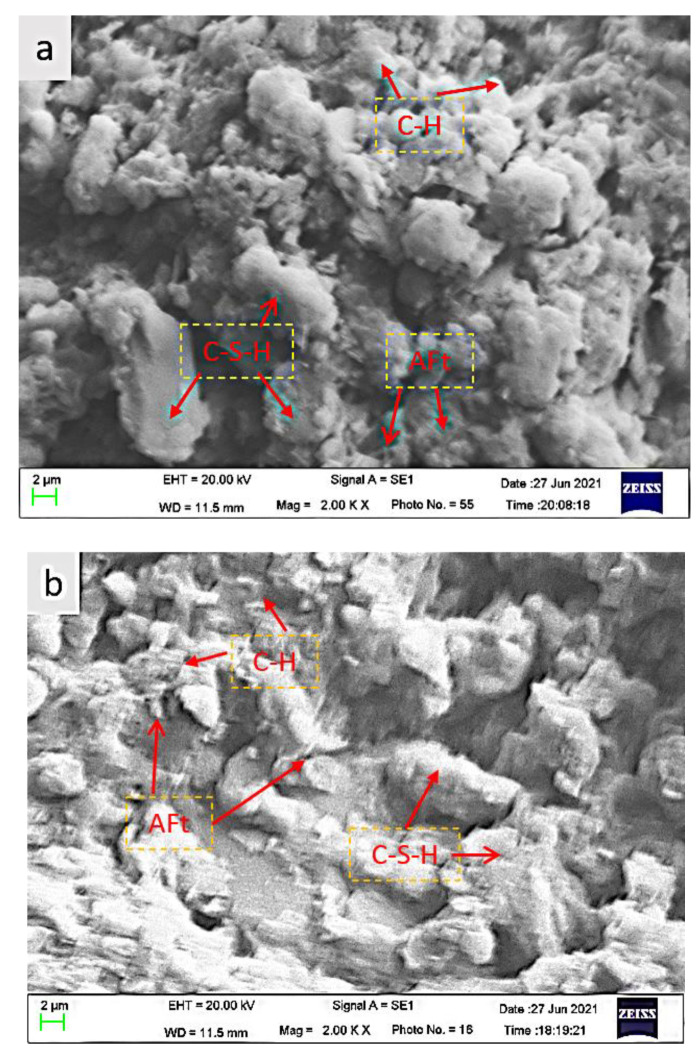
SEM of CFTCB with different curing times: (**a**) 3 d; (**b**) 7 d.

Figure 13 shows the micro-morphology of the CFTCB at different curing times. The picture is not very clear due to operational reasons, but it still shows that numerous precipitates appeared on the surface of the particles after 3 days of curing, and the hydration products were mainly Ca(OH))2 and C-S-H, with a small amount of Aft interspersed. After curing for 7 days, most of the tailing particles were embedded in the matrix of the filling material, the acicular Aft crystal and the layered C-S-H became more obvious, and the structure of the filling material increased in density. Therefore, the generation of hydration products is the main reason for the formation of backfill strength, and the amplitude and speed of strength growth are closely related to the form and quantity of hydration products. Generally, the higher the number of hydration products is, the denser the structure and higher the strength of the backfill, but establishing which hydration products have a greater impact on the strength growth of the backfill requires further study.

### 3.4. Evolution Law and Stage Analysis of Early Intensity

Table 4 shows that under the conditions of a slurry concentration of 64% and CTR of 1:6, the UCS of CFTCB increased significantly with the increase in curing time. After 7 days of curing, the UCS of the specimen doubled compared with that after 2 days of curing. When the slurry concentration and the CTR are the same, the strength of the early backfill increases with the curing time, and the strength of the backfill reaches its maximum on the 7th day of curing. This phenomenon occurs because the generation of hydration products results in the continuous cementation of the particles, so the backfill strength in the early stage increases with the hydration reaction.

**Figure 14:**
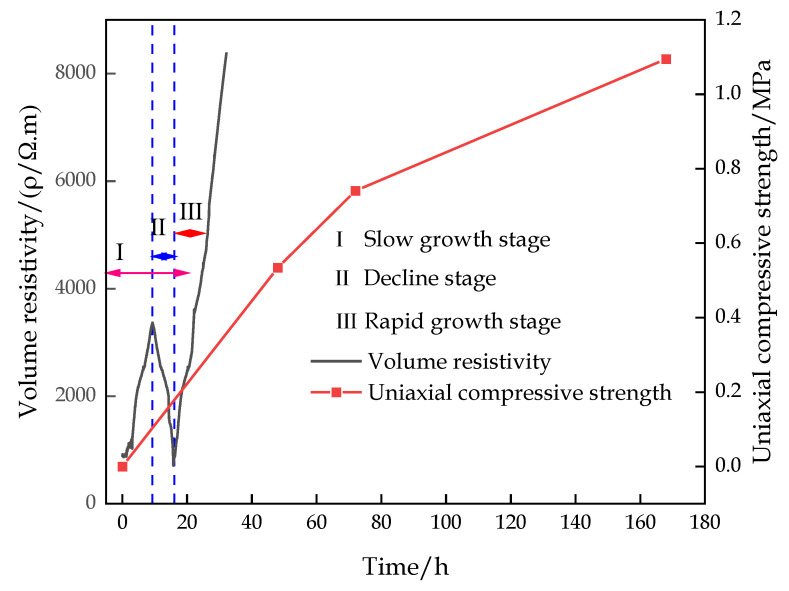
Curve of volume resistivity and UCS over time.

Figure 14 shows the change curve of the volume resistivity and the UCS over time. The figure indicates that within the change period of volume resistivity, the strength of the filling body increases slowly and is unaffected by the volume resistivity, while the volume resistivity gradually increases with the curing days, which is similar to the change law of UCS.

**Figure 15:**
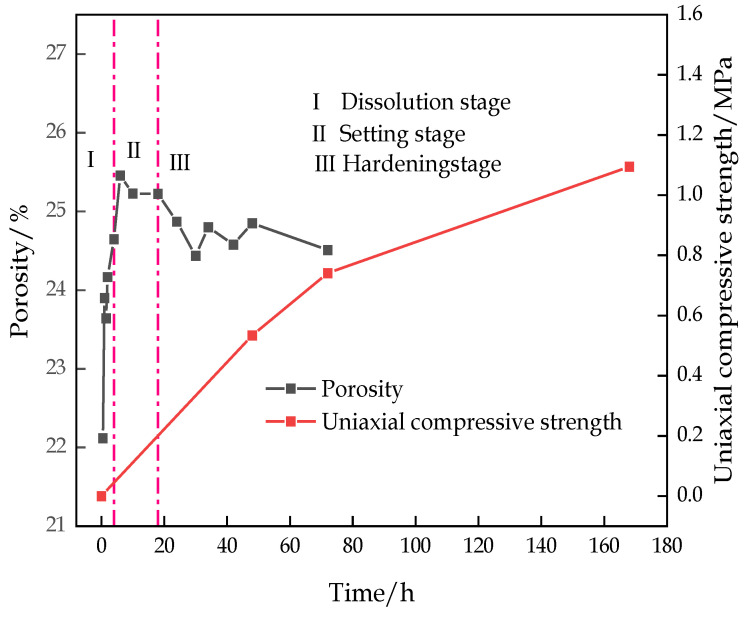
Variation curves of porosity and UCS over time.

Figure 15 shows the variation curve of the porosity and the UCS over time. The figure indicates that the porosity of the sample decreased when the dissolution stage ended, but the backfill had a denser structure, and the strength of the backfill continuously increased with the progress of the hydration reaction.

**Figure 16:**
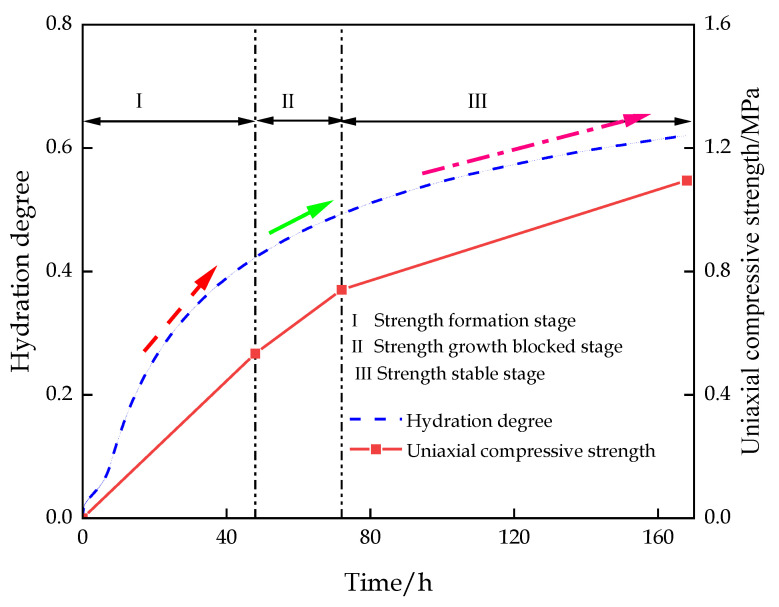
Variation curves of hydration degree and UCS over time.

The CEMHYD 3D hydration model was used to numerically simulate the microstructure evolution in the hydration process. The changes in hydration degree, hydration products, and porosity with hydration time were obtained. The calculated results of the analyzed hydration model were basically consistent with the experimental data. As shown in Figure 16, according to the degree of hydration reaction, the variation in backfill strength can be divided into three stages: strength formation stage I, strength growth blocked stage II, and strength stable stage III. The analysis combined with the stage characteristics of the hydration reaction and the hydration product as follows:

(1) Strength formation stage I

At this stage, after stirring the filling material with water, the main components in the filling material dissolve quickly, and the concentration of Ca^2+^ in the filling slurry increases, resulting in the saturation of Ca(OH)_2_ in the slurry and rapid crystallization. Furthermore, the C-S-H gel is continuously increasing. At this stage, the hydration reaction has reached the hardening stage, and the generation of hydration products is the main reason for the formation and rapid growth of the backfill strength.

(2) Strength growth blocked stage II

At this stage, the hydration reaction decelerates, and the hydration products covering the particles of the filling material block the dissolution of ions, resulting in a reduction in the concentration of ions that can participate in the hydration reaction in the slurry. Consequently, the growth rate of the filling body’s strength decelerates.

(3) Strength stable stage III

At this stage, the hydration reaction stops, and all components involved in the hydration reaction in the cementitious material no longer dissolve. Combined with the analysis of the morphology of hydration products, the result indicates that many hydration products crystallize on the particles of the filling materials, forming a hardened crystalline structure, which leads to the condensation and solidification of filling materials as a whole and stabilizes the strength of the filling body.

## 4. Conclusions

On the basis of the hydration reaction characteristics of the CFTFS, this paper studied the early strength rule and reached the following conclusions. Notably, this study did not consider the influence of the hydration exothermic in the division of the hydration reaction stage, which has certain limitations. Future work with exothermic hydration should be considered to further study the early strength evolution rule of classified fine tailings backfills.

According to the NMR experiment, the porosity of the CFTFS presents a trend of a rapid increase to a slow decrease, and the pore size of the CFTFS, the number of large pores, and the porosity increase with the CTR. In the CFTFS, water exists in the form of adsorbent water; from the perspective of water content, the adsorbed water and free water are converted to each other, the pore water eventually decreases, and the final reduced water content is transformed into chemically bound water that cannot be detected by NMR experiment. The hydration reaction of the CFTFS is divided into three stages, namely, dissolution stage I, setting stage II, and hardening stage III, which explain the characteristics of each hydration process of the CFTFS.

The variation trend of volume resistivity is related to the degree of hydration reaction. In the initial stage of the hydration reaction, the cementation of the sample is dominant, and the volume resistivity of the sample increases slowly. In the middle stage, the resistance and the volume resistivity decrease due to the increase in ion concentration. In the latter stage, the sample slurry gradually condensed with the progress of the hydration reaction, resulting in the gradual increase in the internal resistance of the material, indicating that the resistivity increased with time.

According to the results of the phase analysis and the SEM experiment, the CFTCB condenses and forms a new flake (Ca(OH)_2_), a layered C-S-H, an acicular Aft, and other hydration products with the hydration reaction, increasing the density of the backfill structure and explaining the formation of the early backfill strength.

On the basis of the hydration characteristics of the backfill, hydration products increase the early strength of the backfill, while the reduction in the concentration of ions involved in the hydration reaction will lead to the blockage of the strength growth. Finally, the strength of the backfill tends to stabilize with the completion of the hydration reaction.

## Figures and Tables

**Table 1:** Physical properties of classified fine tailings.

Material Name	Specific Gravity	Loose Density/(g·cm^−1^)	Average Particle Size/µm	Porosity/%
Classified fine tailings	2.69	0.86	44.21	57.36

**Table 2:** Main chemical composition of classified fine tailings.

Chemical Composition	SiO_2_	Al_2_O_3_	CaO	Fe_2_O_3_	K_2_O	MgO
Percentage/%	61.754	12.689	9.182	5.953	4.437	3.305

**Table 3:** Main chemical composition of filling C material.

Chemical Composition	CaO	SiO_2_	Al_2_O_3_	MgO	Fe_2_O_3_	TiO_2_
Percentage/%	44.57	29.95	10.1	6.64	1.25	0.72

**Table 4:** Variation in UCS with curing time.

Cure Time/d	2	3	7
UCS/MPa	0.534	0.741	1.095
